# Contribution of Excessive Alcohol Consumption to Deaths and Years of Potential Life Lost in the United States

**DOI:** 10.5888/pcd11.130293

**Published:** 2014-06-26

**Authors:** Mandy Stahre, Jim Roeber, Dafna Kanny, Robert D. Brewer, Xingyou Zhang

**Affiliations:** Author Affiliations: Jim Roeber, New Mexico Department of Health, Santa Fe, New Mexico; Dafna Kanny, Robert D. Brewer, Xingyou Zhang, Centers for Disease Control and Prevention, Atlanta, Georgia.

## Abstract

**Introduction:**

Excessive alcohol consumption is a leading cause of premature mortality in the United States. The objectives of this study were to update national estimates of alcohol-attributable deaths (AAD) and years of potential life lost (YPLL) in the United States, calculate age-adjusted rates of AAD and YPLL in states, assess the contribution of AAD and YPLL to total deaths and YPLL among working-age adults, and estimate the number of deaths and YPLL among those younger than 21 years.

**Methods:**

We used the Centers for Disease Control and Prevention’s Alcohol-Related Disease Impact application for 2006–2010 to estimate total AAD and YPLL across 54 conditions for the United States, by sex and age. AAD and YPLL rates and the proportion of total deaths that were attributable to excessive alcohol consumption among working-age adults (20-64 y) were calculated for the United States and for individual states.

**Results:**

From 2006 through 2010, an annual average of 87,798 (27.9/100,000 population) AAD and 2.5 million (831.6/100,000) YPLL occurred in the United States. Age-adjusted state AAD rates ranged from 51.2/100,000 in New Mexico to 19.1/100,000 in New Jersey. Among working-age adults, 9.8% of all deaths in the United States during this period were attributable to excessive drinking, and 69% of all AAD involved working-age adults.

**Conclusions:**

Excessive drinking was responsible for 1 in 10 deaths among working-age adults in the United States. AAD rates vary across states, but excessive drinking remains a leading cause of premature mortality nationwide. Strategies recommended by the Community Preventive Services Task Force can help reduce excessive drinking and harms related to it.

## MEDSCAPE CME

Medscape, LLC is pleased to provide online continuing medical education (CME) for this journal article, allowing clinicians the opportunity to earn CME credit.

This activity has been planned and implemented in accordance with the Essential Areas and policies of the Accreditation Council for Continuing Medical Education through the joint sponsorship of Medscape, LLC and Preventing Chronic Disease. Medscape, LLC is accredited by the ACCME to provide continuing medical education for physicians.

Medscape, LLC designates this Journal-based CME activity for a maximum of 1 **AMA PRA Category 1 Credit(s)™**. Physicians should claim only the credit commensurate with the extent of their participation in the activity.

All other clinicians completing this activity will be issued a certificate of participation. To participate in this journal CME activity: (1) review the learning objectives and author disclosures; (2) study the education content; (3) take the post-test with a 75% minimum passing score and complete the evaluation at www.medscape.org/journal/pcd (4) view/print certificate.

**Release date: June 26, 2014; Expiration date: June 26, 2015**

### Learning Objectives

Upon completion of this activity, participants will be able to:

- Analyze different forms of problem drinking in terms of promoting alcohol-attributable deaths and years of potential life lost
- Evaluate the epidemiology of alcohol-attributable deaths in the United States
- Identify the state with the highest rate of alcohol-attributable deaths and associated years of potential life lost
- Estimate the relative mortality burden of alcohol-attributable deaths in the United States

**EDITORS**

Rosemarie Perrin, Technical Writer/Editor, *Preventing Chronic Disease*. Disclosure: Rosemarie Perrin has disclosed no relevant financial relationships.

**CME AUTHOR**

Charles P. Vega, MD, Associate Professor and Residency Director, Department of Family Medicine, University of California, Irvine. Disclosure: Charles P. Vega, MD, has disclosed the following financial relationships: Served as an advisor or consultant for: McNeil Pharmaceuticals

**AUTHORS AND CREDENTIALS**

Disclosures: Mandy Stahre, PhD, MPH; Jim Roeber, MSPH; Dafna Kanny, PhD; Robert Brewer, MD, MSPH; and Xingyou Zhang, PhD, have disclosed no relevant financial relationships.

Affiliations: Mandy Stahre, Washington State Department of Health, Olympia, Washington; Jim Roeber, New Mexico Department of Health, Santa Fe, New Mexico; Dafna Kanny, Robert Brewer, Xingyou Zhang, Centers for Disease Control and Prevention, Atlanta, Georgia.

## Introduction

Excessive alcohol use is the fourth leading preventable cause of death in the United States (1) and costs $223.5 billion, or about $1.90 per drink, in 2006 (2). Excessive alcohol consumption includes binge drinking (ie, ≥5 drinks on an occasion for men; ≥4 drinks on an occasion for women), heavy weekly alcohol consumption (ie, ≥15 drinks/week for men; ≥8 drinks/week for women), and any drinking by pregnant women or those younger than 21 years (2). Binge drinking, the most common form of excessive alcohol consumption, usually results in acute intoxication and is responsible for over half of deaths and three-quarters of the economic costs of excessive drinking. Excessive drinking is also responsible for many other health and social problems (3,4).

In 2004, the Centers for Disease Control and Prevention (CDC) released an online version of the Alcohol-Related Disease Impact (ARDI) application to allow state public health agencies and other users to assess deaths and years of potential life lost (YPLL) attributable to excessive drinking. By using ARDI, CDC estimated approximately 75,000 deaths and 2.3 million YPLL were due to excessive drinking in the United States in 2001 (5). However, since that time, no comprehensive analysis has been conducted of US deaths and YPLL from excessive alcohol consumption. Furthermore, the ARDI application does not provide rates for death and YPLL from excessive drinking. The assessment of these rates is important because the total number of alcohol-attributable deaths (AAD) and YPLL are known to vary substantially across states (6), as does the prevalence and intensity of binge drinking (3). Finally, the contribution of excessive drinking to deaths among working-age adults (20–64 y) and those younger than 21 years is not well understood, even though excessive drinking is known to be a major cause of premature mortality, resulting in an average of 30 years of life lost per AAD (5).

The objectives of this study were to update previous national estimates of AAD and YPLL in the United States, calculate age-adjusted rates of AAD and YPLL in states, assess the contribution of AAD and YPLL to total deaths and YPLL among working-age adults, and estimate the number of deaths and YPLL that specifically involved those younger than 21 years.

## Methods

We estimated average annual deaths and YPLL from 2006 through 2010 that were attributable to excessive drinking by using the CDC’s ARDI online application (6). The methods used in ARDI were developed by a scientific workgroup that comprised experts in alcohol and public health. The details of these methods have been discussed elsewhere (5). Briefly, ARDI estimates AAD by multiplying the number of age- and sex-specific deaths from 54 alcohol-related causes, identified by the underlying cause of death reported on death certificates, by the alcohol-attributable fractions (AAF) for that cause of death.

The majority of AAF for chronic conditions are calculated by ARDI on the basis of relative risk estimates from meta-analyses and the prevalence of alcohol use at specified risk levels (7,8). Self-reported alcohol use from the Behavioral Risk Factor Surveillance System (BRFSS) (9) was used to capture drinking at levels specified by the meta-analyses, which use slightly higher cut-points for risky drinking than those more commonly used in the United States. For the majority of acute conditions (ie, injuries), ARDI includes a direct estimate of the AAF. AAF for these conditions is based on studies assessing the proportion of deaths from a particular condition that occurred at or above a blood alcohol level of 0.10 g/dL (10). In addition, certain conditions (eg, alcoholic cirrhosis of the liver) are by definition 100% alcohol-attributable and therefore did not need to be estimated. To calculate YPLL attributable to excessive alcohol consumption, the age- and sex-specific AAD estimates for each cause were multiplied by the corresponding estimate of life expectancy based on the age and sex of the decedent.

For causes of death that were considered chronic (eg, cancer, liver disease, cardiovascular disease), AAD and YPLL were estimated for decedents aged 20 years or older; for the majority of acute conditions, they were estimated for decedents aged 15 years or older. However, ARDI also estimates AAD and YPLL for chronic conditions for persons younger than 20 years who died from conditions attributable to drinking during pregnancy (eg, fetal alcohol spectrum disorders) and for acute conditions for persons younger than 15 years who died from motor-vehicle traffic crashes or child maltreatment. ARDI provides reports of AAD and YPLL by sex, age group, and state, and for those under age 21 years.

AAD and YPLL due to excessive alcohol use, including those among decedents under age 21 years, were obtained directly from the ARDI application. Average annual national and state rates for AAD and YPLL per 100,000 population from 2006 through 2010 were calculated by dividing the average annual AAD and YPLL estimates from ARDI for 2006 through 2010 by the average annual population estimates from the US Census for 2006–2010, and then multiplying by 100,000. The rates were then age-adjusted to the 2000 US population (11).

The proportion of total average annual deaths and YPLL among working-age adults that were alcohol-attributable was calculated by dividing the average annual AAD and YPLL estimates for adults aged 20 to 64 years from 2006 through 2010 from ARDI by the total average annual deaths and YPLL for all causes for adults aged 20 to 64 years from vital statistics, and then multiplying by 100.

## Results

An average of 87,798 AAD and 2,560,290 YPLL occurred in the United States annually from 2006 through 2010 (Table 1). Overall, 44% of the AAD and 33% of the YPLL were due to chronic conditions, and 56% of the AAD and 67% of the YPLL were caused by acute conditions. Most AAD (71%) and YPLL (72%) involved males. The most common cause of chronic AAD was alcoholic liver disease, while the most common cause of acute AAD was motor-vehicle traffic crashes.

**Table 1:** Average Annual Number of Deaths and Years of Potential Life Lost (YPLL) Attributable to the Harmful Effects of Excessive Alcohol Use, by Cause and Sex, United States, 2006–2010

Cause	Deaths			YPLL		
	Male, n (%)	Female, n (%)	Total	Male, n (%)	Female, n (%)	Total
**Chronic causes**						
Acute pancreatitis	411(57)	313 (43)	724	8,459 (62)	5,263 (38)	13,722
Alcohol abuse	1,587 (78)	435 (22)	2,022	39,949 (76)	12,842 (24)	52,791
Alcohol cardiomyopathy	441 (86)	73 (14)	514	10,357 (84)	1,909 (16)	12,266
Alcohol dependence syndrome	2,892 (78)	836 (22)	3,728	72,208 (75)	24,099 (25)	96,307
Alcohol polyneuropathy	7 (100)	0	7	117 (100)	0	117
Alcohol-induced chronic pancreatitis	59 (72)	23 (28)	82	1,546 (70)	673 (30)	2,219
Alcoholic gastritis	23 (79)	6 (21)	29	586 (75)	191 (25)	777
Alcoholic liver disease	10,403 (72)	3,961 (28)	14,364	251,921 (69)	114,347 (31)	366,268
Alcoholic myopathy	1 (100)	0	1	23 (100)	0	23
Alcoholic psychosis	502 (77)	151 (23)	653	10,511 (76)	3,294 (24)	13,805
Breast cancer (female only)	NA	391 (100)	391	NA	7,429 (100)	7,429
Cholelithiases	0	0	0	0	0	0
Chronic hepatitis	1 (100)	< 1	1	20 (71)	8 (29)	28
Chronic pancreatitis	139 (55)	116 (45)	255	2,940 (56)	2,297 (44)	5,237
Degeneration of nervous system due to alcohol	104 (83)	22 (17)	126	1,804 (79)	477 (21)	2,281
Epilepsy	108 (53)	95 (47)	203	3,170 (55)	2,612 (45)	5,783
Esophageal cancer	437 (89)	55 (11)	492	6,957 (89)	848 (11)	7,805
Esophageal varices	47 (72)	18 (28)	65	1,032 (72)	397 (28)	1,430
Fetal alcohol syndrome	3 (75)	1 (25)	4	163 (68)	78 (32)	241
Fetus and newborn affected by maternal use of alcohol	1 (50)	1 (50)	2	75 (48)	80 (52)	155
Gastro-esophageal hemorrhage	19 (61)	12 (39)	31	332 (66)	173 (34)	505
Hypertension	874 (55)	729 (45)	1,603	13,684 (61)	8,737 (39)	22,421
Ischemic heart disease	516 (70)	223 (30)	738	6,745 (73)	2,434 (27)	9,178
Laryngeal cancer	198 (86)	33 (14)	231	3,126 (84)	581 (16)	3,707
Liver cancer	752 (75)	245 (25)	997	13,033 (77)	3,893 (23)	16,926
Liver cirrhosis, unspecified	4,592 (59)	3,255 (41)	7,847	93,308 (59)	64,114 (41)	157,422
Low birth weight, prematurity, intrauterine growth restriction death	106 (64)	60 (36)	165	7,915 (62)	4,790 (38)	12,705
Oropharyngeal cancer	309 (85)	56 (15)	365	5,401 (86)	912 (14)	6,313
Portal hypertension	24 (63)	14 (37)	38	511 (66)	261 (34)	772
Prostate cancer	202 (100)	NA	202	1,985 (100)	NA	1,985
Psoriasis	<1	<1	<1	2 (67)	1 (33)	3
Spontaneous abortion	NA	<1	<1	NA	10 (100)	10
Stroke, hemorrhagic	1,357 (83)	286 (17)	1,643	21,292 (83)	4,389 (17)	25,681
Stroke, ischemic	329 (74)	118 (26)	447	3,812 (76)	1,227 (24)	5,039
Superventricular cardiac dysrhythymia	122 (43)	160 (57)	282	1,065 (44)	1,356 (56)	2,421
**Subtotal**	**26,564 (69)**	**11,689 (31)**	**38,253**	**584,050 (68)**	**269,722 (32)**	**853,771**
**Acute causes**						
Air–space transport	81 (84)	15 (16)	96	2,408 (81)	569 (19)	2,977
Alcohol poisoning	1,264 (77)	383 (23)	1,647	42,299 (75)	13,833 (25)	56,132
Aspiration	125 (57)	94 (43)	220	2,431 (59)	1,701 (41)	4,132
Child maltreatment	98 (59)	70 (42)	167	6,947 (57)	5,345 (43)	12,292
Drowning	770 (80)	193 (20)	963	27,802 (82)	6,194 (18)	33,997
Excessive blood alcohol level	0	0	0	0	0	0
Fall injuries	3,853 (51)	3,688 (49)	7,541	53,443 (58)	39,015 (42)	92,458
Fire injuries	645 (59)	444 (41)	1,089	15,914 (59)	11,014 (41)	26,928
Firearm injuries	86 (88)	12 (12)	98	3,337 (87)	481 (13)	3,817
Homicide	6,221 (80)	1,535 (20)	7,756	274,753 (81)	64,612 (19)	339,364
Hypothermia	177 (67)	88 (33)	265	4,114 (72)	1,585 (28)	5,699
Motor-vehicle nontraffic crashes	171 (78)	49 (22)	220	5,345 (77)	1,554 (23)	6,899
Motor-vehicle traffic crashes	9,764 (78)	2,696 (22)	12,460	398,376 (77)	121,314 (23)	519,690
Occupational and machine injuries	126 (94)	8 (6)	134	3,359 (94)	201 (6)	3,560
Other road vehicle crashes	146 (79)	38 (21)	184	4,857 (78)	1,363 (22)	6,220
Poisoning (not alcohol)	5,457 (65)	2,947 (35)	8,404	203,635 (65)	111,371 (35)	315,007
Suicide	6,460 (79)	1,719 (21)	8,179	210,811 (77)	62,395 (23)	273,206
Suicide by and exposure to alcohol	28 (67)	14 (33)	42	842 (62)	524 (38)	1,366
Water transport	69 (87)	10 (13)	79	2,349 (85)	427 (15)	2,776
**Subtotal**	**35,540 (72)**	**14,004 (28)**	**49,544**	**1,263,023 (74)**	**443,497 (26)**	**1,706,519**
**Total**	**62,104 (71)**	**25,693 (29)**	**87,798**	**1,847,072 (72)**	**713,218 (28)**	**2,560,290**

Abbreviation: NA, not applicable.

A total annual average of 4,358 AAD (5%) and 249,727 YPLL (10%) involved those under age 21 years from 2006 through 2010 (data not shown). Similar to the findings for adults, about 78% of the AAD and 76% of the YPLL in those younger than 21 involved males. However, in contrast to the findings for adults, all of the top 3 causes of death for those under age 21 years —specifically, motor-vehicle traffic crashes, homicide, and suicide —were acute conditions. In fact, motor-vehicle traffic crashes alone accounted for 36% of the total AAD for those under age 21 years.

The average annual age-adjusted AAD rate for the United States from 2006 through 2010 was 27.9 deaths per 100,000 population, with a range of 51.2 deaths per 100,000 (New Mexico) to 19.1 deaths per 100,000 (New Jersey) (Table 2). Twenty-six states and the District of Columbia (DC) had higher average annual age-adjusted AAD rates than the national rate, and 2 states (New Mexico and Alaska) reported average annual age-adjusted AAD rates above 40 deaths per 100,000 population. The average annual age-adjusted YPLL rate for the United States from 2006 through 2010 was 831.6 per 100,000 population, with a range of 1,570 YPLL per 100,000 (New Mexico) to 570 YPLL per 100,000 (Hawaii) (Table 3). The average annual age-adjusted YPLL rates in 23 states and the District of Columbia were higher than the national rate, and 12 states and DC reported over 1,000 YPLL per 100,000 population.

**Table 2:** Average Annual Number of Deaths and Alcohol-Attributable Deaths (AAD), and Percentage of Deaths Among All Ages and Among Persons Aged 20–64 years, by State, United States, 2006–2010.

State	All Ages				20–64 years		
	Total Deaths	Total AAD	Age-Adjusted AAD Rate per 100,000	Total Alcohol-Attributable Deaths, %	Total Deaths	Total AAD	Total Alcohol-Attributable Deaths, %
United States, total	2,445,322	87,798	27.9	3.6	620,259	60,617	9.8
Alabama	47,377	1,511	31.0	3.2	13,688	1,119	8.2
Alaska	3,531	275	41.1	7.8	1,443	229	15.9
Arizona	46,023	2,362	37.2	5.1	12,178	1,626	13.4
Arkansas	28,600	920	31.0	3.2	7,874	650	8.3
California	234,436	10,572	29.1	4.5	60,612	7,476	12.3
Colorado	30,684	1,628	33.2	5.3	8,429	1,200	14.2
Connecticut	28,794	836	22.1	2.9	5,904	544	9.2
Delaware	7,477	248	26.8	3.3	1,958	172	8.8
District of Columbia	5,035	210	34.7	4.2	1,732	155	9.0
Florida	170,507	6,643	32.6	3.9	40,970	4,493	11.0
Georgia	69,347	2,555	27.6	3.7	21,580	1,854	8.6
Hawaii	9,591	304	20.8	3.2	2,355	191	8.1
Idaho	10,985	437	28.9	4.0	2,578	291	11.3
Illinois	101,218	3,042	23.4	3.0	24,479	2,067	8.4
Indiana	55,816	1,646	25.1	2.9	14,102	1,168	8.3
Iowa	27,682	775	23.8	2.8	5,322	459	8.6
Kansas	24,508	762	26.6	3.1	5,453	518	9.5
Kentucky	40,976	1,351	30.5	3.3	11,518	994	8.6
Louisiana	40,433	1,475	32.8	3.6	12,495	1,103	8.8
Maine	12,534	372	24.8	3.0	2,722	241	8.9
Maryland	43,677	1,318	22.6	3.0	11,928	899	7.5
Massachusetts	52,954	1,525	21.8	2.9	10,920	1,022	9.4
Michigan	87,136	2,945	28.1	3.4	21,977	2,020	9.2
Minnesota	37,897	1,257	23.3	3.3	7,896	778	9.9
Mississippi	28,603	1,025	34.8	3.6	8,711	755	8.7
Missouri	54,990	1,866	30.3	3.4	13,661	1,256	9.2
Montana	8,713	390	37.7	4.5	2,090	275	13.2
Nebraska	15,121	422	22.7	2.8	3,040	261	8.6
Nevada	19,147	943	34.9	4.9	5,979	694	11.6
New Hampshire	10,186	341	23.8	3.3	2,289	222	9.7
New Jersey	69,557	1,754	19.1	2.5	15,543	1,206	7.8
New Mexico	15,670	1,042	51.2	6.6	4,619	758	16.4
New York	147,610	4,011	19.6	2.7	33,826	2,659	7.9
North Carolina	76,780	2,761	28.9	3.6	20,949	1,947	9.3
North Dakota	5,832	179	26.2	3.1	1,123	115	10.2
Ohio	107,798	3,288	26.9	3.1	25,994	2,179	8.4
Oklahoma	36,120	1,350	35.9	3.7	9,974	1,000	10.0
Oregon	31,655	1,302	32.1	4.1	7,456	863	11.6
Pennsylvania	125,482	3,510	25.8	2.8	26,807	2,290	8.5
Rhode Island	9,625	292	25.3	3.0	1,948	188	9.7
South Carolina	40,107	1,534	32.6	3.8	11,995	1,133	9.4
South Dakota	7,003	249	30.0	3.6	1,431	158	11.0
Tennessee	58,120	2,064	31.8	3.6	16,891	1,511	8.9
Texas	162,469	6,514	27.9	4.0	47,458	4,660	9.8
Utah	14,171	529	22.9	3.7	3,751	393	10.5
Vermont	5,170	183	26.5	3.5	1,125	103	9.2
Virginia	58,536	1,865	23.1	3.2	15,193	1,292	8.5
Washington	47,696	1,981	29.2	4.2	11,702	1,301	11.1
West Virginia	21,195	660	33.1	3.1	5,540	468	8.4
Wisconsin	46,442	1,706	28.5	3.7	9,866	1,027	10.4
Wyoming	4,305	210	37.5	4.9	1,188	159	13.4

**Table 3:** Average Annual Number of Years of Potential Life Lost (YPLL), Total YPLL, and Percentage of YPLL Among All Ages and Among Persons Aged 20 to 64 Years, by State, United States, 2006–2010

State	All Ages				20–64 years		
	Total YPLL	Total Alcohol-Attributable YPLL	Age-Adjusted YPLL Rate per 100,000	Total Alcohol-Attributable YPLL, %	Total YPLL	Total Alcohol-Attributable YPLL	Total Alcohol-Attributable YPLL, %
United States, total	38,281,133	2,560,290	831.6	6.7	18,380,927	2,106,126	11.5
Alabama	797,361	48,424	1030.1	6.1	408,573	40,535	9.9
Alaska	75,697	9,131	1299.6	12.1	45,281	8,042	17.8
Arizona	757,615	68,826	1111.8	9.1	368,170	56,603	15.4
Arkansas	469,241	28,226	991.3	6.0	234,355	23,211	9.9
California	3,704,628	304,472	822.0	8.2	1,806,358	251,821	13.9
Colorado	506,006	47,269	942.8	9.3	254,887	40,451	15.9
Connecticut	398,287	23,149	646.4	5.8	173,316	18,988	11.0
Delaware	119,510	7,453	840.5	6.2	58,397	6,079	10.4
District of Columbia	93,741	6,725	1083.9	7.2	52,568	5,426	10.3
Florida	2,580,471	187,068	999.6	7.2	1,217,429	154,447	12.7
Georgia	1,227,003	79,183	829.1	6.5	645,519	65,864	10.2
Hawaii	145,318	7,915	569.7	5.4	68,676	6,335	9.2
Idaho	171,134	12,311	819.7	7.2	76,901	9,873	12.8
Illinois	1,557,893	91,615	711.8	5.9	723,596	73,823	10.2
Indiana	879,690	50,042	780.9	5.7	416,119	41,253	9.9
Iowa	375,846	19,885	654.8	5.3	153,969	15,498	10.1
Kansas	364,862	22,131	792.1	6.1	161,373	18,091	11.2
Kentucky	672,103	41,780	969.0	6.2	341,312	35,393	10.4
Louisiana	715,228	49,719	1116.6	7.0	379,576	41,270	10.9
Maine	176,731	9,929	723.2	5.6	77,630	8,064	10.4
Maryland	713,579	40,075	694.8	5.6	357,601	32,410	9.1
Massachusetts	728,381	41,501	616.0	5.7	318,262	34,389	10.8
Michigan	1,343,335	84,215	838.0	6.3	644,275	68,738	10.7
Minnesota	537,350	32,829	616.2	6.1	231,357	26,237	11.3
Mississippi	504,546	32,916	1134.4	6.5	261,516	27,550	10.5
Missouri	856,379	55,681	941.2	6.5	405,162	44,787	11.1
Montana	133,084	11,331	1163.5	8.5	62,408	9,471	15.2
Nebraska	214,124	11,682	651.0	5.5	88,984	9,037	10.2
Nevada	334,423	27,923	1034.9	8.3	177,069	23,441	13.2
New Hampshire	145,490	8,789	637.1	6.0	66,054	7,260	11.0
New Jersey	1,005,669	50,856	575.8	5.1	457,224	42,068	9.2
New Mexico	268,778	31,129	1570.1	11.6	142,364	26,281	18.5
New York	2,162,819	111,986	564.5	5.2	985,558	90,878	9.2
North Carolina	1,259,703	83,125	886.8	6.6	619,963	68,842	11.1
North Dakota	81,298	5,132	785.5	6.3	33,320	4,061	12.2
Ohio	1,632,999	91,851	789.8	5.6	757,943	74,828	9.9
Oklahoma	595,524	41,460	1134.1	7.0	295,639	34,833	11.8
Oregon	462,860	33,933	868.3	7.3	215,541	27,934	13.0
Pennsylvania	1,789,327	100,106	794.0	5.6	785,357	81,180	10.3
Rhode Island	131,293	7,538	687.4	5.7	56,371	6,178	11.0
South Carolina	680,320	47,267	1037.5	6.9	353,461	39,646	11.2
South Dakota	101,838	7,023	889.3	6.9	42,598	5,519	13.0
Tennessee	972,290	63,058	999.8	6.5	500,315	52,831	10.6
Texas	2,799,886	199,618	823.6	7.1	1,429,308	165,170	11.6
Utah	245,204	16,800	673.9	6.9	119,423	14,075	11.8
Vermont	72,760	4,335	664.6	6.0	32,292	3,317	10.3
Virginia	931,966	55,232	687.3	5.9	447,064	45,349	10.1
Washington	719,348	53,050	784.1	7.4	342,548	43,400	12.7
West Virginia	330,370	19,464	1056.6	5.9	162,457	16,477	10.1
Wisconsin	665,699	44,249	769.0	6.6	289,133	34,776	12.0
Wyoming	72,123	6,480	1183.3	9.0	36,352	5,563	15.3

Average annual AAD were responsible for an average of 9.8% of total deaths (Table 2) and an average of 11.5% of YPLL among working-age adults (20–64 y) (Table 3) from 2006 through 2010.The average proportion of total deaths among working-age adults that were alcohol-attributable ranged from 16.4% in New Mexico to 7.5% in Maryland; the average proportion of total YPLL that were alcohol-attributable ranged from 18.5% in New Mexico to 9.1% in Maryland.

From 2006 through 2010 more than two-thirds (69%) of all average annual AAD (Table 2) and 82% of average annual YPLL (Table 3) involved working-age adults (20–64 y). The proportion of average annual AAD in states that involved working-age adults ranged from 83% in Alaska to 56% in Vermont, and the proportion of average annual YPLL attributable to alcohol that involved working-age adults ranged from 88% in Alaska to 77% in Nebraska and Vermont.

## Discussion

From 2006 through 2010, excessive alcohol consumption accounted for nearly 1 in 10 deaths and over 1 in 10 years of potential life lost among working-age adults in the United States. Furthermore, an average of 2 out of 3 AAD and 8 out of 10 alcohol-attributable YPLL involved working-age adults. Although AAD rates varied by state, the national annual average AAD rate of 27.9 deaths per 100,000 population was higher than the average annual death rate for 10 of the 15 leading causes of deaths from 2006 through 2010 (12). The majority of the average annual AAD involved males (71%); over half of AAD and two-thirds of YPLL resulted from acute causes of death, all of which were by definition attributable to binge drinking. About 5% of all average annual AAD and 10% of average annual YPLL involved those under age 21 years, most of which were due to acute conditions.

The average annual estimates of AAD and YPLL for the United States from 2006 through 2010 are similar to the 2001 estimates (5) and emphasize the substantial and ongoing public health impact of excessive drinking in the United States. The differences in age-adjusted AAD and YPLL rates in states probably reflect differences in the prevalence of excessive drinking, particularly binge drinking, which is affected by state and local laws governing the price, availability, and marketing of alcoholic beverages (13). The differences in AAD and YPLL rates in states probably also reflect other factors, including access to medical care and vehicle miles traveled, which could affect the risk of death from alcohol-related conditions (13,14). The higher rates of AAD and YPLL among men than women probably also reflects the higher prevalence, frequency, and intensity of binge drinking, the most common pattern of excessive alcohol consumption, among men (15).

The substantial contribution of excessive alcohol consumption to total deaths and premature mortality among working-age adults (20–64 y) in the United States, as well as the large proportion of these deaths (69%) and YPLL (82%) that involved working-age adults, is consistent with studies assessing the contribution of harmful alcohol consumption to the global burden of disease (16) and also reflects the substantial effect that excessive alcohol consumption has across the lifespan. The concentration of AAD and YPLL among working-age adults is also a major factor contributing to alcohol-attributable productivity losses from premature mortality, which, together with reduced earnings by excessive drinkers, was responsible for 72% of the estimated $223.5 billion in economic costs from excessive alcohol consumption in 2006 (2).

The findings in this report are subject to several limitations. First, data on alcohol consumption used to calculate indirect estimates of AAF are based on self-reports and may underestimate the true prevalence of excessive alcohol consumption because of underreporting by survey respondents and sampling noncoverage (17). A recent study that used BRFSS data found that self-reports identify only 22% to 32% of presumed alcohol consumption in states on the basis of alcohol sales (18). Second, risk estimates used in ARDI were calculated by using average daily alcohol consumption levels that begin at levels greater than those typically used to define excessive drinking in the United States. Third, deaths among former drinkers, who might have discontinued their drinking because of alcohol-related health problems, are not included in the calculation of AAF, even though some of these deaths might have been alcohol-attributable. Fourth, ARDI does not include estimates of AAD for several causes (eg, tuberculosis, pneumonia, hepatitis C) for which alcohol is believed to be an important risk factor, but for which suitable pooled risk estimates were not available. Fifth, ARDI exclusively uses the underlying cause of death from vital statistics data to identify alcohol-related causes and does not consider contributing causes of death that might be alcohol-related. Finally, age-specific estimates of AAF were only available for motor-vehicle traffic deaths, even though alcohol involvement varies by age, particularly for acute causes of death. While our results do show the substantial burden of alcohol-related consequences, many of the limitations cited could result in a substantial underestimate of the true contribution of excessive alcohol consumption to total deaths and YPLL in the United States.

This analysis illustrates the magnitude and variability of the health consequences of excessive alcohol consumption in the United States, and the substantial contribution of excessive drinking to premature mortality among working-age adults. More widespread implementation of interventions recommended by the Community Preventive Services Task Force (19), including increasing alcohol prices by raising alcohol taxes, enforcing commercial host (dram shop) liability, and regulating alcohol outlet density, could reduce excessive alcohol consumption and the health and economic costs related to it.

## Figures and Tables

**Table:** 

**1. The activity supported the learning objectives.**				
**Strongly Disagree**				**Strongly Agree**
1	2	3	4	5
**2. The material was organized clearly for learning to occur.**				
**Strongly Disagree**				**Strongly Agree**
1	2	3	4	5
**3. The content learned from this activity will impact my practice.**				
**Strongly Disagree**				**Strongly Agree**
1	2	3	4	5
**4. The activity was presented objectively and free of commercial bias.**				
**Strongly Disagree**				**Strongly Agree**
1	2	3	4	5

## Post-Test Information

To obtain credit, you should first read the journal article. After reading the article, you should be able to answer the following, related, multiple-choice questions. To complete the questions (with a minimum 75% passing score) and earn continuing medical education (CME) credit, please go to http://www.medscape.org/journal/pcd. Credit cannot be obtained for tests completed on paper, although you may use the worksheet below to keep a record of your answers. You must be a registered user on Medscape.org. If you are not registered on Medscape.org, please click on the "Register" link on the right hand side of the website to register. Only one answer is correct for each question. Once you successfully answer all post-test questions you will be able to view and/or print your certificate. For questions regarding the content of this activity, contact the accredited provider, CME@medscape.net. For technical assistance, contact CME@webmd.net. American Medical Association's Physician's Recognition Award (AMA PRA) credits are accepted in the US as evidence of participation in CME activities. For further information on this award, please refer to http://www.ama-assn.org/ama/pub/about-ama/awards/ama-physicians-recognition-award.page. The AMA has determined that physicians not licensed in the US who participate in this CME activity are eligible for **AMA PRA Category 1 Credits**™. Through agreements that the AMA has made with agencies in some countries, AMA PRA credit may be acceptable as evidence of participation in CME activities. If you are not licensed in the US, please complete the questions online, print the AMA PRA CME credit certificate and present it to your national medical association for review.
